# Soluble guanylate cyclase stimulator reduced the gastrointestinal fibrosis in bleomycin-induced mouse model of systemic sclerosis

**DOI:** 10.1186/s13075-021-02513-y

**Published:** 2021-05-03

**Authors:** Yuzuru Yamamoto, Takaichi Okano, Hirotaka Yamada, Kengo Akashi, Sho Sendo, Yo Ueda, Akio Morinobu, Jun Saegusa

**Affiliations:** 1 Department of Rheumatology and Clinical Immunology, Kobe University Graduate School of Medicine, Kobe, Japan; 2 Department of Clinical Laboratory, Kobe University Hospital, Kobe, Japan

**Keywords:** Systemic sclerosis, Gastrointestinal fibrosis, Soluble guanylate cyclase stimulator

## Abstract

**Background:**

Systemic sclerosis (SSc) is a chronic autoimmune-mediated connective tissue disorder. Although the etiology of the disease remains undetermined, SSc is characterized by fibrosis and proliferative vascular lesions of the skin and internal organs. SSc involves the gastrointestinal tract in more than 90 % of patients. Soluble guanylate cyclase (sGC) stimulator is used to treat pulmonary artery hypertension (PAH) and has been shown to inhibit experimental skin fibrosis.

**Methods:**

Female C57BL/6J mice were treated with BLM or normal saline by subcutaneous implantation of osmotic minipump. These mice were sacrificed on day 28 or day 42. Gastrointestinal pathologies were examined by Masson Trichrome staining. The expression of fibrosis-related genes in gastrointestinal tract was analyzed by real-time PCR, and the levels of collagen in the tissue were measured by Sircol collagen assay. To evaluate peristaltic movement, the small intestinal transport (ITR%) was calculated as [dyeing distance × (duodenum − appendix)] − 1 × 100 (%). We treated BLM-treated mice with sGC stimulator or DMSO orally and analyzed them on day 42.

**Results:**

Histological examination revealed that fibrosis from lamina propria to muscularis mucosa in the esophagus was significantly increased in BLM-treated mice, suggesting that BLM induces esophageal hyperproliferative and prefibrotic response in C57BL/6J mice. In addition, the gene expression levels of Col3a1, CCN2, MMP-2, MMP-9, TIMP-1, and TIMP-2 in the esophagus were significantly increased in BLM-treated mice. More severe hyperproliferative and prefibrotic response was observed in the mice sacrificed on day 42 than the mice sacrificed on day 28. The ITR% was found to be significantly lower in BLM-treated mice, suggesting that gastrointestinal peristaltic movement was reduced in BLM-treated mice. Furthermore, we demonstrated that sGC stimulator treatment significantly reduced hyperproliferative and prefibrotic response of esophagus and intestine in BLM-treated mice, by histological examination and Sircol collagen assay.

**Conclusions:**

These findings suggest that BLM induces gastrointestinal hyperproliferative and prefibrotic response in C57BL/6J mice, and treatment with sGC stimulator improves the BLM-induced gastrointestinal lesion.

## Background

Systemic sclerosis (SSc) is a connective-tissue disease of unknown etiology. SSc is characterized by autoimmunity, microvascular impairment, chronic inflammation, and fibrotic changes in various organs [1]. There are several mouse models of SSc which develop dermal thickening and fibrosis, the most obvious feature of human SSc. However, they exhibit only some aspects of the disease or develop additional abnormalities not associated with SSc in humans [2].

Bleomycin (BLM) is a chemotherapeutic agent that is used in the management of some human malignancies such as lymphomas and squamous cell carcinomas. The major limitation of BLM therapy is pulmonary toxicity and skin fibrosis [3]. On the other hand, BLM-treated mice are widely accepted as an experimental model of SSc and are mainly used for estimating skin fibrosis. Lee et al. recently reported that systemic delivery of BLM using osmotic minipumps caused lung fibrosis from a peripheral lung lesion, which is similar to the lung fibrosis observed in human SSc patients [4].

On the other hand, more than 90% of SSc patients develop gastrointestinal tract fibrosis. The fibrosis extends from the mouth to the anus, and the esophagus and anorectum are most frequently affected [5, 6]. The esophageal fibrosis causes a reduced quality of life in SSc patients due to gastroesophageal reflux disease and decreased peristaltic movement. Nevertheless, only symptomatic treatment is currently available for gastrointestinal manifestations in SSc [6]. In addition, there have been few reports of mouse models that reproduce the gastrointestinal lesions of SSc [5, 7]. An epithelial Fli1-deficient mouse has been reported to develop skin, lung, and esophagus fibrosis [8]. The transgenic mouse strain TβRIIδk-fib is characterized by ligand-dependent upregulation of TGFβ signaling and has been shown to develop skin and lung fibrosis. This TG mouse model was previously shown to develop colonic fibrosis [9]. However, there have been no reports about gastrointestinal lesion in BLM-treated mice.

A soluble guanylate cyclase (sGC) stimulator is known as a drug for treatment of pulmonary arterial hypertension in SSc patients. This treatment stimulates soluble guanylate cyclase, increasing cyclic guanosine monophosphate (cGMP) levels and activating protein kinase G (PKG) in the cytosol, resulting in subsequent relaxation of vascular smooth muscle cells. In addition, recent studies have shown that sGC stimulator dose-dependently inhibits the fibrosis of kidney, skin, liver, and intestine in several mouse models, thus playing a critical role in fibrotic disease [10]. For example, sGC stimulator reduces skin and intestinal fibrosis in experimental sclerodermatous chronic graft-versus-host-disease (Scl-GvHD) [11].

In this study, we provide the first demonstration that continuous subcutaneous administration of BLM induced gastrointestinal fibrosis in mice, which histologically resembled human SSc. In addition, peristaltic movement was significantly impaired in the mice. Furthermore, we revealed that treatment with an sGC stimulator ameliorated gastrointestinal lesion in the esophagus and intestine of BLM-treated mice.

## Materials and methods

### Animals

Female C57BL/6J mice aged 8 to 9 weeks old were obtained from CLEA Japan, Inc. (Osaka, Japan). We used the mice from 9 to 10 weeks of age. These mice were housed in the animal facility of Kobe University, with a 12-h dark/light cycle at a constant temperature, and were provided with food and water ad libitum. All procedures were carried out in accordance with the recommendations of the Institutional Animal Care Committee of Kobe University.

### Reagents and antibodies

BLM was purchased from Nippon Kayaku (Tokyo, Japan). Dimethyl sulfoxide (DMSO) and 2 M acetic acid were purchased from Sigma–Aldrich (St. Louis, MO, USA). A soluble guanylate cyclase stimulator (BAY 63-2521) was purchased from Selleck Chemicals (Houston, TX, USA). Sircol collagen assay was purchased from Biocolor Ltd. (Belfast, UK). Alzet mini-osmotic pumps model 2001 were purchased from Durect Corporation (Cupertino, CA, USA). Disposable oral tubes (disposable feeding needle FG5202) were purchased from Fuchigami Corporation (Kyoto, Japan). RNeasy Mini kits were purchased from Qiagen (Tokyo, Japan). For immunohistochemistry experiments, anti-alpha smooth-muscle actin (αSMA) antibody (ab124964 EPR5368), anti-proliferating cell nuclear antigen (PCNA) antibody (ab92552 EPR3821), and rabbit monoclonal IgG (ab172730 ERP25A) were purchased from Abcam (Cambridge, UK). A rabbit ABC Staining System (sc-2018) was purchased from Santa Cruz Biotechnology (Dallas, TX, USA).

### BLM administration

BLM was dissolved in normal saline (NS). BLM or NS were administered with osmotic minipumps, as described in previous reports, with minor modifications [4] [12, 13]. The osmotic minipumps containing 200 μL of BLM (125 mg/kg) or NS were implanted subcutaneously under the loose skin on the backs of C57BL/6J mice on day 0. The pumps delivered 1.0 mg/h for 7 days. The mice were euthanized on day 28 (4 w) or on day 42 (6 w).

For the sGC stimulator treatment experiment, we removed these pumps on day 7, and then administered 200 μL daily DMSO or sGC stimulator orally to mice from day 14 to day 42. The mice were sacrificed on day 42 (6 w), and gastrointestinal lesions were harvested.

### Histology

The esophageal and intestinal samples were fixed in 4 % paraformaldehyde, embedded in paraffin, sectioned, and stained with Masson’s trichrome (MT). To evaluate the esophageal and intestinal fibrosis caused by BLM treatment, we measured the thickness between the top of the fibrosis layer and the muscularis mucosa at × 40 magnification under a BZ-X700 fluorescence microscope (Keyence, Osaka, Japan). We analyzed the full length of the esophagus and the intestine up to 5 cm from the pylorus.

### Immunohistochemistry and morphometric analysis

Tissue array blocks were cut into 4 mm-thick sections, which were deparaffinized in a dry oven at 60 °C for 1 h. To detect αSMA and PCNA immunoreactivity, samples were immersed in Target Retrieval Solution (Dako) and incubated at 95 °C for 30 min for antigen retrieval. Endogenous peroxidase activity was blocked with 0.3 % hydrogen peroxide for 30 min. After protein blocking for 1 h, the samples were incubated with a 1:50 dilution of rabbit monoclonal anti-αSMA antibody and PCNA antibody overnight at 4 °C. The secondary antibody was then applied with the ImmunoCus ABC Staining System (Santa Cruz Biotechnology), according to the manufacturer’s protocol. We examined under a BZ-700 All-in-one Microscope (Keyence, Osaka, Japan). For morphometric analysis, photographs were taken of at least 10 different fields at each mouse (n = 6-10) with a BZ-700 All-in-one Microscope. The αSMA positive area in the esophagus was measured with Dynamic cell count BZ-X Image Converter (Keyence). PCNA-positive cells in the esophagus and intestine were counted at × 200 magnification [14].

### Changes in body weight

We evaluated the change in body weight of the mice from day 0 to day 28 or day 42. The change was calculated using the following formula:

$$ \mathrm{Body}\ \mathrm{weight}\ \mathrm{change}\ \left(\%\right)=\left[\left(\mathrm{body}\ \mathrm{weight}\ \mathrm{on}\ \mathrm{day}\ 28\ \mathrm{or}\ \mathrm{on}\ \mathrm{day}\ 42\right)-\left(\mathrm{body}\ \mathrm{weight}\ \mathrm{on}\ \mathrm{day}\ 0\right)\right]\times 1/\left(\mathrm{body}\ \mathrm{weight}\ \mathrm{on}\ \mathrm{day}\ 0\right)\times 100\ \left(\%\right). $$

### Gastrointestinal transit

We examined the small intestinal transport rate (ITR%) as an indicator of gastrointestinal movement [15]. First, the mice were fasted overnight but given free access to water. Two hundred microliters of Evans blue solution (5 % w/v in NS) was then orally administered to each mouse and dye distance was evaluated. All animals were sacrificed 30 min after Evans blue solution administration, and the rate of gastrointestinal transit was calculated by dividing the distance of the Evans blue migration by the total length of the small intestine. Specifically, the full length of intestine from the pylorus to the ileocecum and the length between the pylorus and the forefront of the transported dye (distance of dye movement) were measured. The ITR% was calculated using the following formula.

$$ \mathrm{Small}\ \mathrm{intestinal}\ \mathrm{transport}\ \mathrm{rate}\ \left(\%\right)\ \left(\mathrm{ITR}\%\right)=\left[\mathrm{distance}\ \mathrm{of}\ \mathrm{dye}\ \mathrm{movement}\ \left(\mathrm{cm}\right)/\mathrm{total}\ \mathrm{length}\ \mathrm{of}\ \mathrm{small}\ \mathrm{intestine}\ \left(\mathrm{cm}\right)\right]\times 100. $$

### Quantitative real-time polymerase chain reaction (rt-PCR)

We examined gene expression in the esophagus and intestine by qPCR to indicate fibrotic gene expression. Total RNA was isolated from the esophagus and intestine using an RNeasy Mini kit purchased from Qiagen, and complementary DNA was reverse-transcribed using a QuantiTect Reverse Transcription kit (Qiagen). PCR reaction mixtures were prepared using the QuantiTect SYBR Green PCR kit (Qiagen). The results were indicated on a PikoReal system. The following primer pairs were used: glyceraldehyde-3-phosphate dehydrogenase (GAPDH), 5′-AACTTTGGCATTGTGGAAG-3′ (forward) and 5′-ACACATTGGGGGTAGGAACA-3′ (reverse); collagen 3a1 (COL3A1), 5′-CAAGGTCTTCCTGGTCAGCCT-3′ (forward) and 5′-TGCCACCAGGAGGAGATCCATCTC-3′ (reverse); cellular communication network factor 2 (CCN2), 5′-CACTCCGGGAAATGCTCCATGTTG-3′ (forward) and 5′-GTTGGGTCTGGGCCAAATGT-3′ (reverse); interleukin-6 (IL-6), 5′TTCCATCCAGTTGCCTTCTTG-3′ (forward) and 5′-TCATTTCCACGATTTCCCAGAG-3′ (reverse); α smooth muscle actin (αSMA), 5′-AGAGACTCTCTTCCAGCCATC-3′ (forward) and 5′-ACACATTGGGGGTAGGAACA-3′ (reverse); collagen 1a1 (COL1A1), 5′-TGACTGGAAGAGCGGAGAGTACT-3′ (forward) and 5′-GGTCTGACCTGTCTCCATGTTG-3′ (reverse); lysyl oxidase (LOX), 5′-GAGTACCGCATCAGCAAAAG-3′ (forward) and 5′-CCCTCCGATTCCATAGTTCAC-3′ (reverse); an alfa-submit of prolyl hydroxylase (P4HA3), 5′-ATGGAAATGGACCCACCAA-3′ (forward) and 5′-TGCAGCCATTATCCTGTGTC-3′ (reverse); tissue inhibitor of metalloproteinases 1 (TIMP1), 5′-GGAAAGCCTCTGTGGATATG-3′ (forward) and 5′-AACAGGGAAACACTGTGC-3′ (reverse); tissue inhibitor of metalloproteinases 2 (TIMP2), 5′-TTCCGGGAATGACATCTATGG-3′ (forward) and 5′-GGGCCGTGTAGATAAACTCGAT-3′ (reverse). GAPDH was used as the internal control to normalize the amount of loaded, complementary DNA (cDNA).

### Measurement of soluble collagen content

Sircol collagen assay (Biocolor Ltd, Belfast, UK) was used to quantify soluble collagen content of the esophagus and intestine. Briefly, we measured the weight of the esophagus and intestine, and then homogenized each tissue. We mixed the homogenate with 100 mL of acid-neutralizing reagent, and 200 mL of cold isolation and concentration reagent, and then added 1 mL of Sircol dye reagent, mixed and allowed to stand for 30 min. After centrifugation, the pellets were dissolved in 750 mL of ice-cold acid salt wash regent and 250 mL of Sircol alkali reagent and vortexed. Relative absorbance was measured at 540 nm.

### Statistical analysis

Data are presented as mean ± standard deviation (SD). Differences between groups were analyzed by unpaired *t* test, one-way ANOVA, and Tukey’s multiple comparison test using GraphPad Prism 5 software (GraphPad Software Inc., La Jolla, CA, USA).

## Results

### BLM caused esophageal and intestinal hyperproliferative and prefibrotic response

**Fig. 1:**
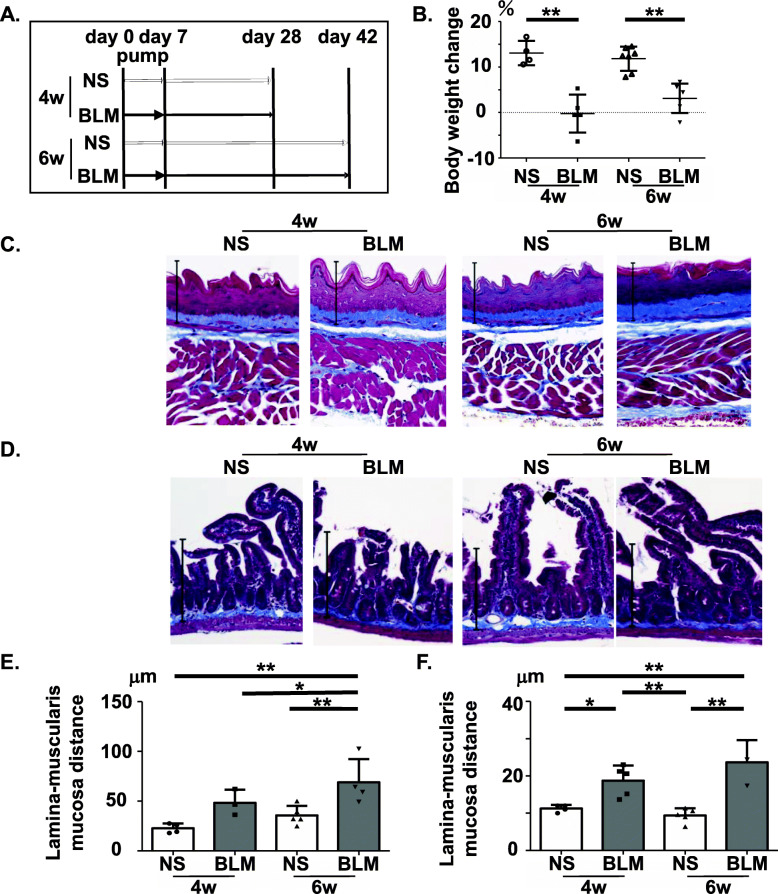
Bleomycin (BLM) caused esophageal and intestinal hyperproliferative and prefibrotic response. **a** Schematic representation of the experimental protocol. Osmotic pumps containing 200 μL of BLM (125 mg/kg) or NS were implanted subcutaneously onto the backs of C57BL/6 mice on day 0. The pumps delivered their contents at a rate of 1.0 μg/h for 7 days. These mice were then sacrificed on day 28 (4 w) or day 42 (6 w). **b** Body weight change from day 0 to day 28 or day 42 in C57BL/6 mice. The body weight change was calculated as [(body weight on day 28 or day 42) − (body weight on day 0)] × (body weight on day 0)^−1^ × 100(%). Each dot indicates the body weight change of an individual mouse. **c**, **d** Representative images of esophageal (**c**) and intestinal (**d**) sections stained with Masson’s trichrome (MT) at × 40 magnification (**c** the straight line represents 100 μm; **d** the straight line represents 200 μm). **e**, **f** The thickness (lamina–muscularis mucosa distance) of esophageal (**e**) and intestinal (**f**) fibrotic tissue stained with MT (*n* = 5–7 mice per group). Bars represent mean + SD. *P < 0.05, **P < 0.01; one-way ANOVA, Tukey’s multiple comparison test. NS, normal saline; BLM, bleomycin

We first evaluated whether BLM induced gastrointestinal fibrosis in female C57BL/6J mice. We administered BLM (125 mg/kg) or NS by subcutaneous implantation of an osmotic mini-pump on day 0 and sacrificed the mice on day 28 (4 w) or day 42 (6 w) (Fig. 1a). BLM-treated mice exhibited decreased body weight (Fig. 1b), but no mice died as a result of the implantation itself or the drug administration. We found that BLM treatment increased the distance from the top of the lamina propria to the muscularis mucosa in the esophagus and intestine (Fig. 1c–f). These results confirmed that BLM-treated mice had significantly increased esophageal and intestinal hyperproliferative and prefibrotic response compared with NS-treated mice. Additionally, we showed that more severe esophageal hyperproliferative and prefibrotic response was observed in the mice sacrificed at day 42 compared to those sacrificed at day 28. Also, we confirmed that BLM treatment caused lung and skin fibrosis in mice as previously reported (data not shown). However, we found no evidence of colon fibrosis in this mouse model (data not shown).

### BLM induced fibrotic gene expression in murine esophagus

**Fig. 2:**
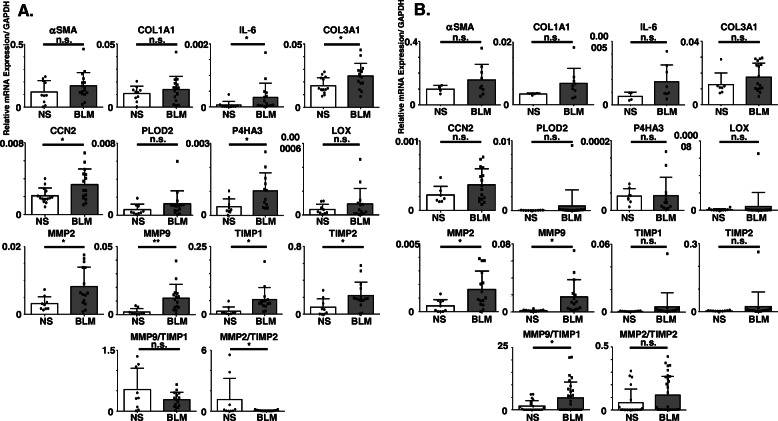
BLM induced fibrotic gene expression in the murine gastrointestinal lesions. Osmotic pumps containing 200 μL of BLM (125 mg/kg) or NS were implanted subcutaneously onto the backs of C57BL/6 mice on day 0. These mice were sacrificed on day 42 (6 w). Real-time PCR determined gene expression levels in the esophagus (**a**) and intestine (**b**) from BLM- or NS-treated mice. Bars represent mean + SD. *P < 0.05, **P < 0.01. n. s. = not significant; unpaired *t* test (*n* = 14–15 mice per group). αSMA, alpha smooth muscles actin; COL1A1, collagen 1a1; IL-6, interleukin-6; COL3A1, collagen 3a1; CCN2, cellular communication network factor 2; P4HA3, an alpha-submit of prolyl hydroxylase; MMP-2, matrix metalloproteinase-2; MMP-9, matrix metalloproteinase-9; TIMP-1, tissue inhibitor of metalloproteinases 1; TIMP-2, tissue inhibitor of metalloproteinases 2

Next, we evaluated fibrotic gene expression in murine esophagus and intestine. The gene expression levels of COL3A1, CCN2 [16], IL-6, P4HA3, MMP-2, MMP-9, TIMP-1 and TIMP-2 in the esophagus were significantly increased in BLM-treated mice compared with NS-treated mice. The ratio of MMP-2/TIMP-2 was significantly decreased in BLM-treated mice compared with NS-treated mice (Fig. 2a). These results indicated that BLM treatment caused esophageal prefibrotic response, as reflected in gene expression levels.

We also evaluated gene expression levels of the intestine. The gene expression levels of MMP-2 and MMP-9 in the intestine were significantly increased in BLM-treated mice compared with NS-treated mice (Fig. 2b).

### BLM treatment reduced the peristaltic distance of the upper gastrointestinal tract

**Fig. 3:**
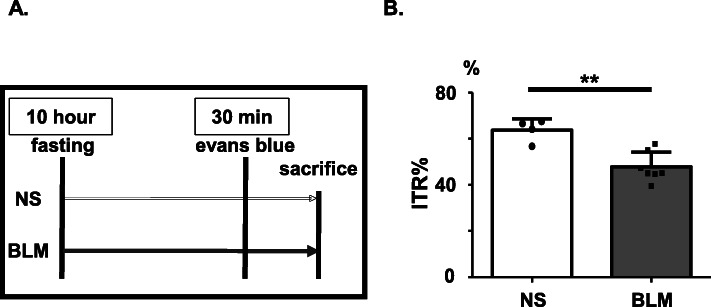
BLM reduced the peristaltic distance of the upper gastrointestinal tract in mice. **a** Schematic representation of the experimental protocol. Osmotic pumps containing 200 μL of BLM (125 mg/kg) or NS were implanted subcutaneously onto the backs of C57BL/6 mice on day 0. On day 42 (6 w), the mice were fasted overnight but given free access to water. Next day, the mice were orally administered 200 μL of Evans blue solution (5 % w/v in NS) and sacrificed 30 min later. The rate of gastrointestinal transit was calculated by dividing the distance of the Evans blue migration by the total length of the intestine. **b** ITR% in BLM- or NS- treated C57BL/6 mice (*n* = 5–7 mice per group). Bars represent mean + SD. **P < 0.01; Unpaired *t* test. ITR%, intestinal transit rates

Most SSc patients have decreased peristaltic movement of the intestine, leading to chronic intestinal pseudo-obstruction (CIPO). Consequently, we next investigated the effect of BLM on peristaltic movement in mice. We analyzed the ITR% to evaluate peristalsis in the upper gastrointestinal tract (Fig. 3a). We sacrificed all mice on day 42 in this experiment. The ITR% of mice treated with BLM was significantly reduced compared to that of NS-treated mice (**P = 0.002) (Fig. 3b). This result suggested that BLM-induced gastrointestinal fibrosis impaired peristaltic movement of gastrointestinal involvement. Our results showed that BLM caused gastrointestinal hyperproliferative and prefibrotic response both histologically and functionally, resembling the gastrointestinal lesions of SSc patients.

### sGC stimulator treatment improved esophageal and intestinal hyperproliferative and prefibrotic response

**Fig. 4:**
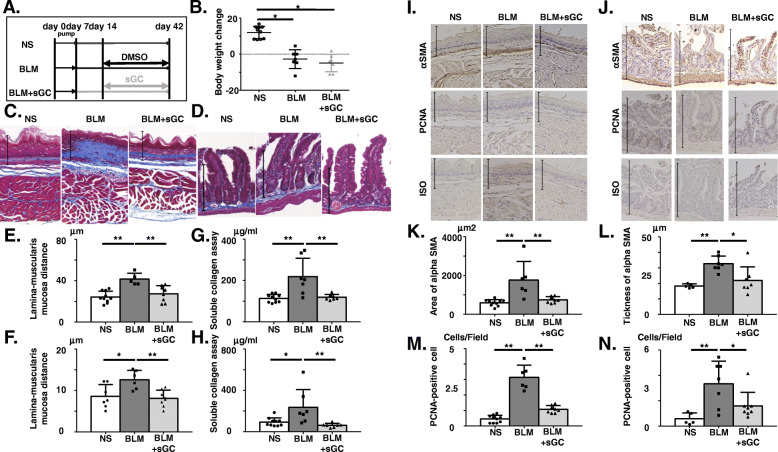
Treatment with a soluble guanylate cyclase (sGC) stimulator improved esophageal and intestinal hyperproliferative and prefibrotic response. **a** Schematic representation of the experimental protocol. Osmotic pumps containing 200 μL of BLM (125 mg/kg) or NS were implanted on day 0. Pumps were removed on day 7. Mice were then treated with an oral sGC stimulator, BAY 63-2521, (10 mg/kg) (BLM + sGC group) or DMSO (BLM group) from day 14 to day 42, and then sacrificed on day 42 (6 w). **b** Body weight change from day 0 to day 42 in C57BL/6 mice. The body weight change was calculated as [(body weight on day 42) − (body weight on day 0)] × (body weight on day 0)^−1^ × 100 (%) (*n* = 6–10 mice per group). **c**, **d** Representative histology of esophagus (**c**) and intestine (**d**) stained with MT at × 40 magnification. **e**, **f** The thickness (lamina–muscularis mucosa distance) of fibrosis in the esophagus (**e**) and intestine (**f**) fibrotic tissues stained with MT (*n* = 6–10 mice per group). **g**, **h** Soluble collagen production in the esophagus (**g**) and intestine (**h**) in NS-, BLM-, and BLM plus sGC stimulator-treated mice (*n* = 6–10 mice per group). **i**, **j** Representative histology of immunostaining with anti-αSMA and anti-PCNA antibodies in NS-, BLM-, and BLM plus sGC stimulator-treated mice (*n* = 6–10 mice per group) in the esophagus (**i**) and intestine (**j**). **k** The area stained positive for anti-αSMA antibody in the esophagus. **l** The thickness of αSMA-positive area in intestine. **m**, **n** The number of PCNA-positive cell in the esophagus (**m**) and intestine (**n**) (**c**, **i** the straight line represents 100 μm. **d**, **j** the straight line represents 200 μm). Bars represent mean + SD. *P < 0.05, **P < 0.01; one-way ANOVA, Tukey’s multiple comparison test. sGC, soluble guanylate cyclase; DMSO, dimethyl sulfoxide; PCNA, proliferating cell nuclear antigen

We next examined the effect of methyl (4, 6-diamino-2-(1-(2-fluorobenzyl)-1H-pyrazolo[3, 4-b] pyridine-3-yl) pyrimidin-5-yl) (methyl) carbamate; Riociguat, BAY 63-2521, a stimulator of sGC, which is a drug used to treat pulmonary arterial hypertension in SSc patients. We administered DMSO or BAY 63-2521 orally to BLM-treated mice. The study design of the sGC-stimulation experiment is described in Fig. 4a. No mice died as a result of the experimental procedures. BLM treatment caused a decrease in body weight, while BAY 63-2521 had no effect (Fig. 4b). We found that histological fibrosis was significantly reduced in the esophagus of BAY 63-2521-treated mice compared to DMSO-treated mice (Fig. 4c, e). We next measured soluble collagen content of the esophagus and intestine by Sircol collagen assay. BLM significantly increased the soluble collagen content of the esophagus, and BAY 63-2521 significantly decreased the soluble collagen content induced by BLM (Fig. 4g). In addition, we confirmed that BAY 63-2521 treatment decreased the thickness from the top of the lamina propria to the muscularis mucosa and the soluble collagen content in the intestine of BLM-treated mice (Fig. 4d, f, h).

We next performed immunohistochemistry staining of mice esophagus and intestine. We measured the area and the thickness of αSMA-positive region and counted the number of PCNA-positive cells in the esophagus and intestine of BLM-treated mice. We found that BLM-administration significantly increased the αSMA-positive area in the esophagus, and BAY 63-2521 treatment significantly decreased the BLM induced αSMA positive area (Fig. 4i, k). The number of PCNA positive cells was significantly increased in the esophagus of BLM-treated mice and was significantly decreased with BAY 63-2521 treatment (Fig. 4m). In addition to the esophagus, the expansion of the αSMA-positive area induced by BLM and its improvement with BAY treatment were also observed in intestine (Fig. 4j, l). The number of PCNA-positive cells of BLM was significantly increased in intestine and significantly decreased with BAY 63-2521 (Fig. 4n). These results indicated that oral sGC-stimulation therapy ameliorated the gastrointestinal hyperproliferative and prefibrotic response induced by BLM. However, sGC stimulation did not significantly improve ITR% (data not shown).

## Discussion

In this study, we provided the first evidence that BLM caused gastrointestinal hyperproliferative and prefibrotic response, demonstrated both histologically and functionally. The gastrointestinal lesions of SSc patients included esophageal dysmotility, lower esophageal sphincter insufficiency, gastroesophageal reflux, esophageal stricture, a reduction in motility in the intestine, wide-mouthed diverticula in the large intestine, and rectal atonia in advanced cases [17]. BLM-induced gastrointestinal lesions resembled several patterns of gastrointestinal lesions observed in SSc patients. In particular, we found that esophageal hyperproliferative and prefibrotic response induced by BLM in mice were similar to the esophageal lesions of SSc patients in terms of increasing thickness of the muscularis mucosa observed by MT staining.

BLM causes inflammation by activating the TGFβ and p53 pathways. These activations result in proliferation of fibroblasts and induce apoptosis of epithelial cells, which in turn cause fibrosis. Previously, BLM has been reported to cause intestinal inflammation by increasing tumor necrosis factor α (TNFα), lipopolysaccharide (LPS), and IL-1β [18]. In our experiments, the gene expression level of IL-6 in the esophagus of BLM-treated mice was significantly increased compared with those of NS-treated mice. This inflammation is a pre-requisite for the initiation of fibrotic lesions [19]. In this mouse model, BLM administration caused an increase in the thickness of gastrointestinal hyperproliferative and prefibrotic response and reduced peristaltic distance. The gene expression levels of COL3A1, CCN2, and P4HA3 in the esophagus of BLM-treated mice were significantly increased compared with those of NS-treated mice. Mishra et al. previously reported that esophageal fibrosis affected the area from the lamina propria to the muscularis mucosa of trichrome-stained paraffin-embedded esophagus in mice and human [20]. Therefore, we measured the fibrotic thickening between the top of the lamina propria and the muscularis mucosa after histopathological staining. Additionally, colon fibrosis is an important part of gastrointestinal fibrosis. A dreaded complication in individuals with SSc is CIPO, which is characterized by bowel dilatation and abnormal motility, and colon fibrosis is an important cause of chronic intestinal failure in patients with SSc [21]. Although fibrosis in the colon is an important feature of gastrointestinal lesions in humans with SSc, we found no evidence of colon fibrosis in this mouse model (data not shown).

We showed that more severe esophageal hyperproliferative and prefibrotic response was observed in the mice sacrificed on day 42 compared to those sacrificed on day 28. However, the severity of inflammation during the onset of fibrogenesis did not correlate with collagen deposition in another model of intestinal fibrosis [19]. Lee et al. previously reported that the lung fibrosis induced by BLM spontaneously decreased after more than 6 weeks [4]. We considered that the period after inflammation might be particularly important. For these reasons, we decided to sacrifice the mice on day 42; however, this might not have been enough time to observe colon fibrosis.

In this study, the gene expression levels of αSMA, Cola1, and PLOD2 tended to be increased in the esophagus of BLM-treated mice on day 42, but no significant difference was observed. If the esophagus was completely fibrotic, the expression of these genes would be increased. Therefore, on day 42, we might have assessed the phase of hyperproliferative and prefibrotic response, which occur before complete fibrosis. On the other hand, a histopathological examination by M&T staining showed that the esophagus of BLM-treated mice had significant fibrotic change compared with the control group on day 42. In addition, immunohistological studies showed a significant increase in αSMA expression in BLM-treated mice. Based on these results, we regarded the changes in the gastrointestinal tract by BLM administration as the hyperproliferative phase as a pre-stage of fibrosis.

In previous reports, animal models of intestinal fibrosis were classified into seven categories: spontaneous, gene-targeted, chemical-, immune-, bacteria-, and radiation-induced as well as postoperative fibrosis [22]. However, we were unable to find a suitable mouse model for human SSc which caused fibrosis in the esophagus and intestine simultaneously after chronic inflammation. For example, the dextran sodium sulfate (DSS) -induced intestinal fibrosis model mouse is the easiest and the most reproducible protocol to induce colonic inflammation with associated fibrosis. However, in this mouse model, fibrosis is induced after acute chemical injury and no esophageal lesion has been documented [22]. Another model, the TGFβ1-overexpression mouse, develops colonic fibrosis with obstruction. However, the intestinal fibrosis of this mouse is focal [22]. There have not been any reports of a mouse model in which esophageal and intestinal fibrosis are caused simultaneously. In our mouse model, it is significant that the esophagus and the intestine both exhibit fibrotic changes at the same time.

sGC promotes production of cGMP. The effect of cGMP is mediated by several downstream targets, including PKG [10]. The de novo synthesis of collagen type I is reduced by sGC due to the inhibition of TGFβ-induced ERK1/2 signaling in human lung fibroblasts [23]. In previous reports, treatment with the sGC stimulator Riociguat, improved the histological fibrosis and hydroxyproline content in intestine compared with control in Scl-GVHD mice. TGFβ plays a central role in fibrosis in Scl-GVHD mice [11, 24]. We demonstrated that BLM induced gastrointestinal hyperproliferative and prefibrotic response in mice. TGFβ1 is known to be involved in BLM-induced organ fibrosis, and activation of TGFβ1 causes fibroblast proliferation [25]. There are several common mechanisms by which cGMP elevation can elicit anti-fibrotic effects. First, cGMP elevation inhibits TGFβ-induced ECM production. Second, cGMP elevation inhibits TGFβ-induced fibroblast to myofibroblast differentiation. Third, cGMP elevation inhibits TGFβ-induced cell proliferation [10, 26]. As sGC stimulation inhibited gastrointestinal hyperproliferative and prefibrotic response in our BLM-treated mice, TGFβ1 may also be involved in BLM-induced gastrointestinal lesions. In our experiments, the gene expression levels of TGFβ1 in the esophagus of BLM-treated mice were significantly increased compared with those of NS-treated mice. Also, the gene expression levels of TGFβ in the esophagus of sGC stimulation-treated mice tended to be lower than those of BLM-treated mice (data not shown).

On the other hand, Hemnes et al. recently reported that PKG activity was decreased by BLM exposure in the lung [27]. sGC stimulation might increase PKG activation in lesions exposed to BLM, which may have been responsible for the therapeutic effect. However, we do not consider that sGC stimulation alone provided the perfect treatment. In fact, sGC stimulation did not significantly improve ITR% (data not shown). These may have been affected by the duration of sGC stimulant treatment.

In an immunohistological examination, we demonstrated that BLM increased the expression of αSMA and PCNA and that sGC stimulator administration decreased their expression. We considered that the elevated expression of αSMA suggests the fibrotic change in tissue, and the increased expression of PCNA suggests an increase in cell proliferation of fibroblasts. It was speculated that the decrease of αSMA and PCNA expression by sGC stimulator treatment was due to the suppression of cell proliferation of fibroblasts. A previous study demonstrated that elevation of sGC suppresses proliferation and survival of human breast cancer cells [28]. The authors showed that overexpression of sGCα1 and sGCα1 in MDA-MB-231 cells suppressed cell proliferation, induced apoptosis, and disturbed cell cycle progression and that sGC induced G1 and G2 cell cycle arrest in the cells. In another report on renal fibrosis, 3-(4,5-dimethylthiazol-2-yl)-2,5-diphenyltetrazolium bromide (MMT) assay revealed that sGC activator significantly suppressed cell proliferation of mesangial cells [29]. It was also revealed that activation of the sGC-sGMP cascade resulted in suppression of hyperproliferation of mesangial cells and decreased the levels of pro-fibrotic markers [29]. Therefore, cell growth inhibitory effects of sGC might be involved in the suppression of BLM-induced gastrointestinal hyperproliferative and prefibrotic response in this study.

The limitation of this study was that not all fibrosis markers were significantly increased in the esophagus and intestine of BLM-treated mice. Based on the results, we did not conclude that fibrotic changes in gastrointestinal tract of BLM-treated mice were complete fibrosis, but regarded them as hyperproliferative and prefibrotic response. On the other hand, histopathological examination by M&T staining confirmed fibrotic changes, and immunohistological examinations also demonstrated an increase in fibrotic markers. These discrepancies have not been explained and need to be investigated in the future.

Riociguat (BAY 63-2521) is known to improve pulmonary arterial hypertension associated with connective tissue diseases (PATENT-1 and PATENT-2) [30]. However, it remains unknown whether Riociguat improves organ fibrosis in SSc. In the RISE-SSc trial and a pilot study, Riociguat did not show a significant effect on the cutaneous lesions of SSc [31, 32]. There has been no report of the effect of Riociguat on gastrointestinal involvement of SSc patients partly because intestinal lesions are difficult to evaluate. Our mouse model may be a simple and usable model to study the gastrointestinal hyperproliferative and prefibrotic response of SSc.

## Conclusion

This study demonstrated that BLM induced gastrointestinal hyperproliferative and prefibrotic response which was ameliorated by sGC stimulation. Our model may be a novel mouse model of gastrointestinal hyperproliferative and prefibrotic response in C57BL/6J mice corresponding to human systemic sclerosis.

## Data Availability

Not applicable.

## Abbreviations

αSMA: Alpha smooth muscles actin
BLM: Bleomycin
cDNA: Complementary deoxyribonucleic acid
cGMP: Cyclic guanosine monophosphate
CIPO: Chronic intestinal pseudo-obstruction
COL1A1: Collagen 1a1
COL3A1: Collagen 3a1
CCN2: Cellular communication network factor 2
DMSO: Dimethyl sulfoxide
DSS: Dextran sodium sulfate
GAPDH: Glyceraldehyde-3-phosphate dehydrogenase
IL-1β: Interleukin-1β
IL-6: Interleukin-6
ITR%: Intestinal transit rates
LOX: Lysyl oxidase
LPS: Lipopolysaccharide
MT: Masson’s trichrome
MMP-2: Matrix metalloproteinase-2
MMP-9: Matrix metalloproteinase-9
NS: Normal saline
PAH: Pulmonary artery hypertension
PCNA: Proliferating cell nuclear antigen
PKG: Protein kinase G
P4HA3: An alfa-submit of prolyl hydroxylase
TGFβ: Transforming growth factor β
TIMP1: Tissue inhibitor of metalloproteinases 1
TIMP2: Tissue inhibitor of metalloproteinases 2
TNFα: Tumor necrosis factor α
Scl-GvHD: Sclerodermatous chronic graft-versus-host-disease
sGC: Soluble guanylate cyclase
SSc: Systemic sclerosis
